# Variation of Anthocyanin Content and Profile Throughout Fruit Development and Ripening of Highbush Blueberry Cultivars Grown at Two Different Altitudes

**DOI:** 10.3389/fpls.2019.01045

**Published:** 2019-09-04

**Authors:** Anna Spinardi, Gabriele Cola, Claudio Sebastiano Gardana, Ilaria Mignani

**Affiliations:** ^1^Department of Agricultural and Environmental Sciences – Production, Landscape, Agroenergy, Università degli Studi di Milano, Milan, Italy; ^2^Department of Food, Environmental and Nutritional Sciences, Università degli Studi di Milano, Milan, Italy

**Keywords:** blueberry, bioactive compounds, ripening class, polyphenols, anthocyanins, altitude, temperature

## Abstract

Blueberry (*Vaccinium corymbosum L.*) is a widely consumed fruit and a rich source of bioactive compounds, namely, the polyphenol class of anthocyanins. Little information is available about the influence of internal (genetic and developmental) and external (environmental) factors on the levels of phenolic metabolites in blueberry fruit. In light of this consideration, total polyphenolic and flavonoid content, anthocyanin accumulation and composition were evaluated in cv. “Duke” and “Brigitta” grown at two different altitudes in Valtellina, a valley of the Alps in Northern Italy. During berry ripening, there is a developmentally coordinated shift from cyanidin-type, di-substituted anthocyanins toward delphinidin-based, tri-substituted pigments. At the lower altitude location, higher temperatures, not exceeding optimum, resulted in a more quickly berry developmental pattern and in higher anthocyanin concentrations in the early phases of ripening. At later stages of ripening, berries of both cultivars at higher altitude compensate for these initial temperature effects, and no differences were recorded in ripe fruit grown in the two locations. We conclude that anthocyanin accumulation is strongly regulated by development and genotype, and the environmental factors, associated to the altitude gradient, exert in the trial conditions only a fine-tuning influence. Fruits reach the full-ripening stage simultaneously at both sites because the initial gap in pigment levels is counterbalanced at the higher altitude by a faster rate of accumulation at later phases of the ripening process.

## Introduction

The consumer’s concern for fruit quality is, at the present, becoming greater due to increasing interest in food aspects related to health care and better quality of life. For these reasons, the modern concept of quality combines sensorial features and nutritional contents (Knee, 2002; Tromp, 2005). According to this trend, small fruit industry is increasing because their high contents in bioactive compounds meet the consumer expectations of healthy food (Gosch, 2003; Kähkäonen et al., 2003).

Blueberry is an interesting fruit for its potential health benefits attributed to antioxidant properties of bioactive compounds (polyphenols, in particular anthocyanins, and ascorbic acid); thus, the worldwide production of blueberry has been growing in the last decades. In particular, blueberry consumption has been reported to induce improvements in memory and cognitive performance (Williams et al., 2008), to prevent oxidative stress and damage, to inhibit inflammation (Youdim et al., 2002), and to improve cardiovascular health (Erlund et al., 2008; Beccaro et al., 2004). Moreover, small fruit cultivation is proper for hill field because of its attitude to mountain climate, aside from lands, and generally in small-scale farms giving an extra income to family businesses and the opportunity to improve the economy of marginal areas. Finally, blueberry production is rapidly increasing because of its excellent productivity, adaptability to different environments, and pest resistance. Moreover, in the recent years, the small fruit production was often oriented to environmental friendly agricultural methods as integrated pest management or organic agriculture, endowing the growing areas with an added value of environmental respect.

In order to better understand the drivers of nutraceutical traits in blueberry, it will be challenging to study the different growing conditions and techniques (Häkkinen and Törrönen, 2000) to assess fruit quality. The content of nutraceutical substances is influenced by ripening stage, genotype, cultivation techniques, pre-harvest climatic conditions, and the operations carried out during the post-harvest storage. The environmental factors play a crucial role in qualitative and quantitative accumulations of antioxidant compounds in many types of fruits: temperature, solar radiation, water stress, and soil are considered the major elements affecting anthocyanin content in fruit (Connor et al., 2002; Castellarin et al., 2007b; Stevenson and Scalzo, 2012). Acidity and phenolic compounds vary markedly from southern to northern latitudes (Åkerström et al., 2010; Thomas et al., 2013, Lätti et al., 2008, Lätti et al., 2010), while the altitudinal gradient induces higher accumulation of anthocyanins in bilberry (Jovančević et al., 2011; Wang et al., 2014). Among the climatic parameters affected by altitude, as sunlight spectra, visible light, and UV radiation, the lower daily temperatures at higher altitudes seem to increase anthocyanin accumulation in bilberry and blueberry (Karppinen et al., 2016; Zoratti et al., 2015a, Zoratti et al., 2015b). The direct sun exposure enhances the expression of flavonoid pathway genes and the concentrations of anthocyanins, catechins, flavonols, and hydroxycinnamic acids in bilberry leaves (Jaakola et al., 2004) and fruit (Jovančević et al., 2011), giving support for the protective role of flavonoids and hydroxycinnamic acids against high solar radiation in plants (Routray and Orsat, 2011). In Finnish bilberry, the proportions of anthocyanins vary in the berries from South compared to those in the Central and Northern regions (Åkerström et al., 2010; Lätti et al., 2008).

According to these considerations, the present work studies the effect of the different altitude on quality attributes of two cultivars of blueberry grown in Valtellina, a Northern Italy valley. Particular attention was focused on quantitative and qualitative changes in anthocyanin accumulation during berry development, as anthocyanins are key factors of quality in various fruits. Fruit colors depending on anthocyanin types, and their contents, have both commercial and aesthetic values.

## Materials and Methods

### Fruit Sampling and Quality Trait Measurement

Highbush blueberry (*Vaccinium corymbosum* L.) samples of two cultivars (“Brigitta” and “Duke”) were harvested in the Valtellina area (Northern Italy) during 2015 and 2016 from two commercial orchards located in Postalesio (46,175° N; 9,776° E) and Gaggio di Berbenno (46,168° N; 9,746° E) at two different altitudes (450 and 650 m ASL, respectively). An East–West exposure characterizes Valtellina, a valley in the Italian Alps. Potential Yearly Photosynthetically Active Radiation (PPAR) was calculated by means of the topographic tools of SAGA GIS (Conrad et al., 2015), based on a 20-m resolution digital elevation model.

Both the orchards were endowed with micro-irrigation systems and monitoring of water status, to avoid summer water stress and grant the correct water supply.

Postalesio soil was characterized by sandy texture class (sand> 61%), sub acidic pH (6.04), and high organic matter content (4.66%). The soil of Gaggio was characterized by sandy texture class (sand >71%), acidic pH (4.98), and high organic matter content (3.15%). Both the soils were optimal for blueberry growth, without differences for nutrient content. Conventional farming practices were carried out in the two fields.

Samples were collected from 4- to 5-year-old plants. “Duke” is an early ripening variety, harvested in Northern Italy at middle June—first week of July, whereas “Brigitta,” a mid-season variety, ripens at middle July—first week of August. “Duke” berries were harvested on the third week of June, and “Brigitta” berries were sampled on the third week of July, in two consecutive growing seasons (2015 and 2016).

**Figure 1:**
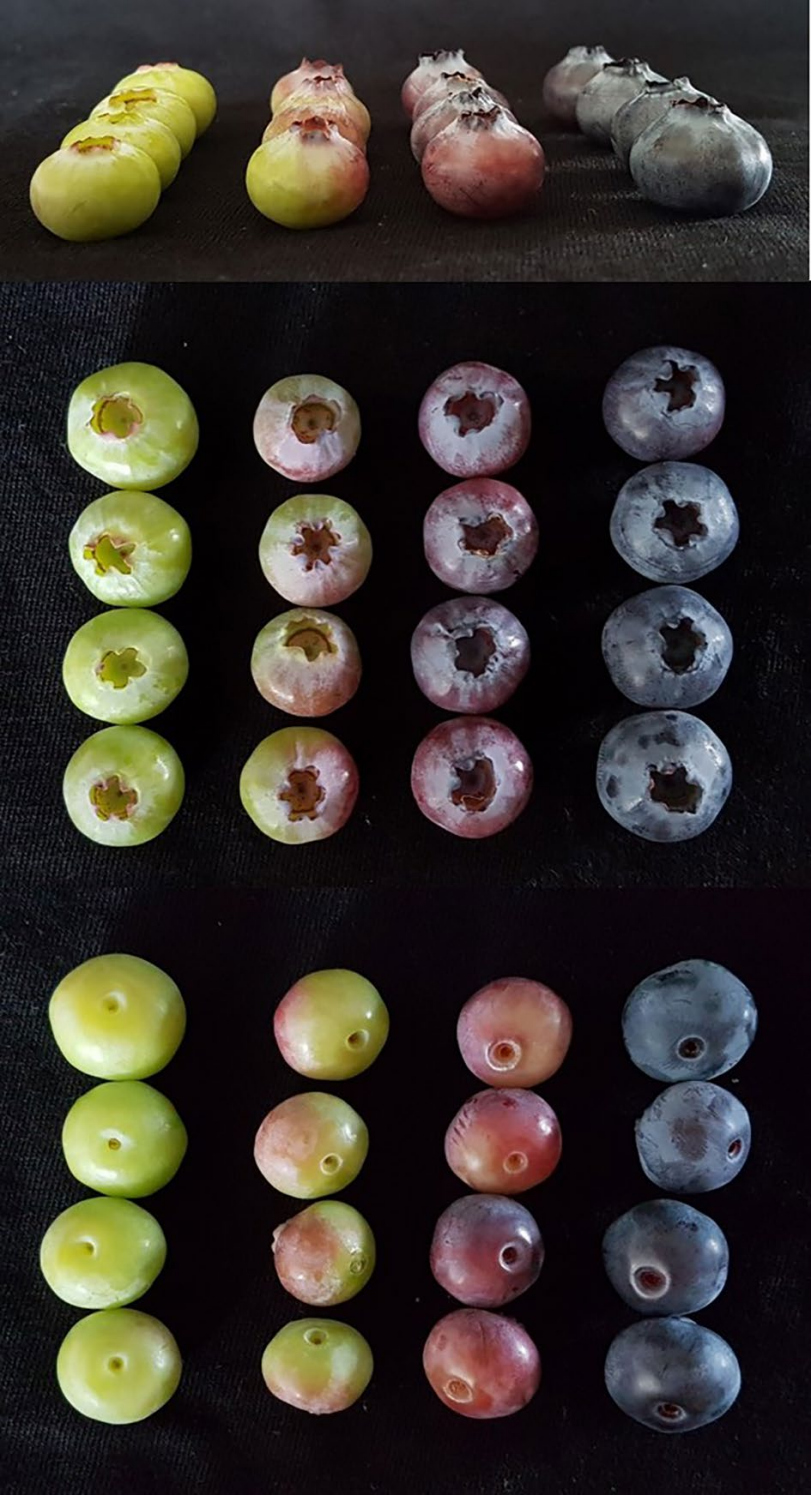
Different ripening stages of blueberry fruit (from left, stage 1: mature green, stage 2: less than 50% pigmented, stage 3: 50% to completely pigmented except the stem-end, stage 4: fully ripe).

The trial considered six plants of each cultivar. All fruits of each bush were picked and divided visually into four homogenous classes corresponding to different stages of ripening: mature green, fully expanded (class I), less than 50% pigmented (class II), 50% to completely pigmented except the stem-end (class III), and fully ripe (class IV) (**Figure 1**). Immature fruits not fully enlarged, i.e., that had not reached at harvest the typical final shape and size due to a complete cell expansion, were discarded. Fruits of each of the four different ripening stages of a single plant represented one individual sample. Consequently, the pool of berries harvested from the same plant at the assigned stage of ripening represented one biological replicate for each cultivar, and the total number of samples was 48 (4 ripening stages per 6 individual plants per 2 cultivars) for each of the growing sites, located at 450 and 650 m ASL, respectively. Total soluble solid content (TSS) and titratable acidity (TA) were also evaluated to better characterize the different ripening stages (**Supplementary Figure 1**). TSS content, expressed as percent of soluble solids, was determined by a hand refractometer (Atago mod., N1, Tokyo, Japan) on juice obtained by squeezing the berries. TA was measured in an aliquot of the juice (about 1 g) through titration to pH 8.1 by 0.01 N NaOH and a semi-automated Titrator Compact D (Crison Instruments SA, Alella, Spain). Acidity was calculated and reported as meq/L. Aliquots of fruits from each sample were used to perform all the analyses. Distance between individual plants allowed optimal illumination of all sides of the canopy. Immediately after harvest and sorting, samples were cooled to below 8°C and placed within the day at −80°C until chemical analyses.

### Temperature Determination

The air temperature of the two sites was directly monitored by two ONSET HOBOware (HOBO, Onset Computer Corporation, Pocasset, MA, USA) experimental weather station equipped with air temperature sensors and solar radiation shields. Daily maximum and minimum temperature and thermal excursion are presented.

### Fruit Skin Color Measurement

Fruit skin color was measured only in 2015 by a compact tristimulus colorimeter (Minolta CR-300, Ramsey, NJ, USA) with an 8-mm diameter viewing aperture and described by the CIE L*, a* and b* color space coordinates. (Dussi et al., 1995). L* represents the lightness of colors on a scale of 0 to 100 and is low on the scale for dark colors and high for bright colors. The parameter a* is negative for bluish-green and positive for red-purple. The parameter b* is negative for blue and positive for yellow. The L*, a* and b* values were used to calculate the hue angle (H° = arctan [b*/a*], reported as degrees) and the chroma index (C* = [a^*2^ + b^*2^]^1/2^) (McGuire, 1992). Hue angle, denoting visual color appearance, was expressed on a 360° color wheel where 0° and 360° represent red-purple, 90° the yellow, 180° the bluish-green, and 270° the blue. Chroma is the degree of deviation from gray towards pure chromatic color and thus indicates color intensity or saturation (high values are more vivid). Color measurements were assessed on 30 freshly harvested fruit per cultivar and maturity stage at each growing location, amounting to 480 determinations.

### Total Phenolics, Total Flavonoids, and Total Anthocyanin Analysis

For total phenolics, total flavonoids, and total anthocyanin determinations, 25 g of berries were ground in 25 mL of acidified ethanol (EtOH:H_2_O:HCl conc.; 70:29:1; v/v/v) and placed 14 h on a rotary shaker, then centrifuged at 10,000 *g* for 10 min. The supernatant was analyzed after proper dilution with acidified ethanol. Six different samples per cultivar (2)/ripening stage (4)/altitude (2) were extracted, accounting for 96 samples.

Total phenolic contents were determined following the Folin–Ciocalteu procedure. One milliliter of Folin–Ciocalteau reagent, 0.5 ml of distilled water, and 2 ml of 20% Na_2_CO_3_ were added to 0.1 mL of the extract. The solution was immediately diluted to a final volume of 20 mL with distilled water and then agitated. The optical density was measured after 90 min at 700 nm on a UV–vis spectrophotometer (Jasko model 7800, Tokyo, Japan). Results were expressed as mg of catechin per g of fresh weight (FW).

Total flavonoids were evaluated spectrophotometrically at 280 nm. A catechin standard curve was set, and results were expressed as milligrams of catechin per g of FW (Beghi et al., 2013).

Total anthocyanin contents of blueberry extracts were estimated spectrophotometrically according to Sinelli et al. (2008) as malvidin 3-glucoside at 520 nm using a molar absorptivity coefficient of 28,000 and reported as mg per g of FW.

### Anthocyanin Identification and Quantification

Approximately 10 g of frozen berries were mixed with 30 mL of a solution methanol/TFA 2% in water (10:90, v/v) and homogenized by an Ultra-Turrax (IKA-Werke, Staufen, D) for 1 min. The homogenate was extracted for 30 min under agitation in the dark at room temperature. The suspension was centrifuged at 1,000 × *g* for 10 min at 4°C, and the supernatant recovered. The residue was extracted again until disappearance of the red color (4 × 20 mL) with a solution of methanol/TFA 2% in water (10:90, v/v), and treated as described above. The supernatants were combined, and the volume was adjusted to 200 mL by solution of 2% TFA in water. All extracts were stored at −20°C and centrifuged at 3,000 × *g* for 1 min before the LC analysis.

Anthocyanin identification was performed using an ACQUITY UHPLC system (Waters, Milford, MA) equipped with a model E-Lambda photodiode array detector (Waters) and an Exactive high-resolution mass spectrometer (Thermo Scientific, Rodano, Italy) equipped with an HESI-II probe for ESI and a collision cell (HCD). A C18 Kinetex column (150 × 4.6 mm, 2.6 μm, Phenomenex, Torrence, CA) protected with guard column, carried out the separation at 1.7 mL/min, and flow-rate split 5:1 before electrospray ionization (ESI) source. The column and sample were maintained at 45^o^ and 20°C, respectively. The eluents were (A) 0.2% TFA in water and (B) acetonitrile: 0.2% TFA in water (35:65, v/v). The linear gradient was as follows: 0–15 min 14% B; 15–25 min from 14% to 20% B; 25–35 min from 20 to 32% B; 35–45 min from 32% to 50% B; 45–48 min 50% to 90% B; and 90% for 3 min. The MS operative conditions were as follows: spray voltage +4.0 kV, sheath gas flow rate 60 (arbitrary units), auxiliary gas flow rate 20 (arbitrary units), capillary temperature 350°C, capillary voltage +30 V, tube lens +80 V, skimmer +25 V, and heater temperature 130°C.

The acquisition was assessed in the full-scan mode in the range (m/z) + 200–2,000 u, using an isolation window of ±2 ppm. The AGC target, injection time, mass resolution, energy, and gas in the collision cell were 1 × 106, 100 ms, 50 K, 20 V, and N_2_, respectively. The MS data were processed using Xcalibur Software (Thermo Scientific). Peaks were identified by evaluating the accurate mass, the fragments obtained in the collision cell, and the on-line UV spectra (220–700 nm). Working solutions (n = 5) were prepared in the range of 2–50 µg/mL, and 20 µL was injected into the chromatographic system. Chromatographic data were integrated at 520 nm, and each analysis was carried out in triplicate (three technical replicates).

### Statistical Analysis

Analysis of variance was performed by IBM SPSS Statistics software, version 25 (SPSS Inc., Chicago, IL), using general linear model univariate analysis, with growing year, altitude, or cultivar as fixed factors. Significant differences among different ripening classes were calculated by Tukey’s mean test. Differences at p ≤ 0.05 were considered as significant. Additional information is reported in the figure legends.

## Results

### Environmental Factors

**Figure 2:**
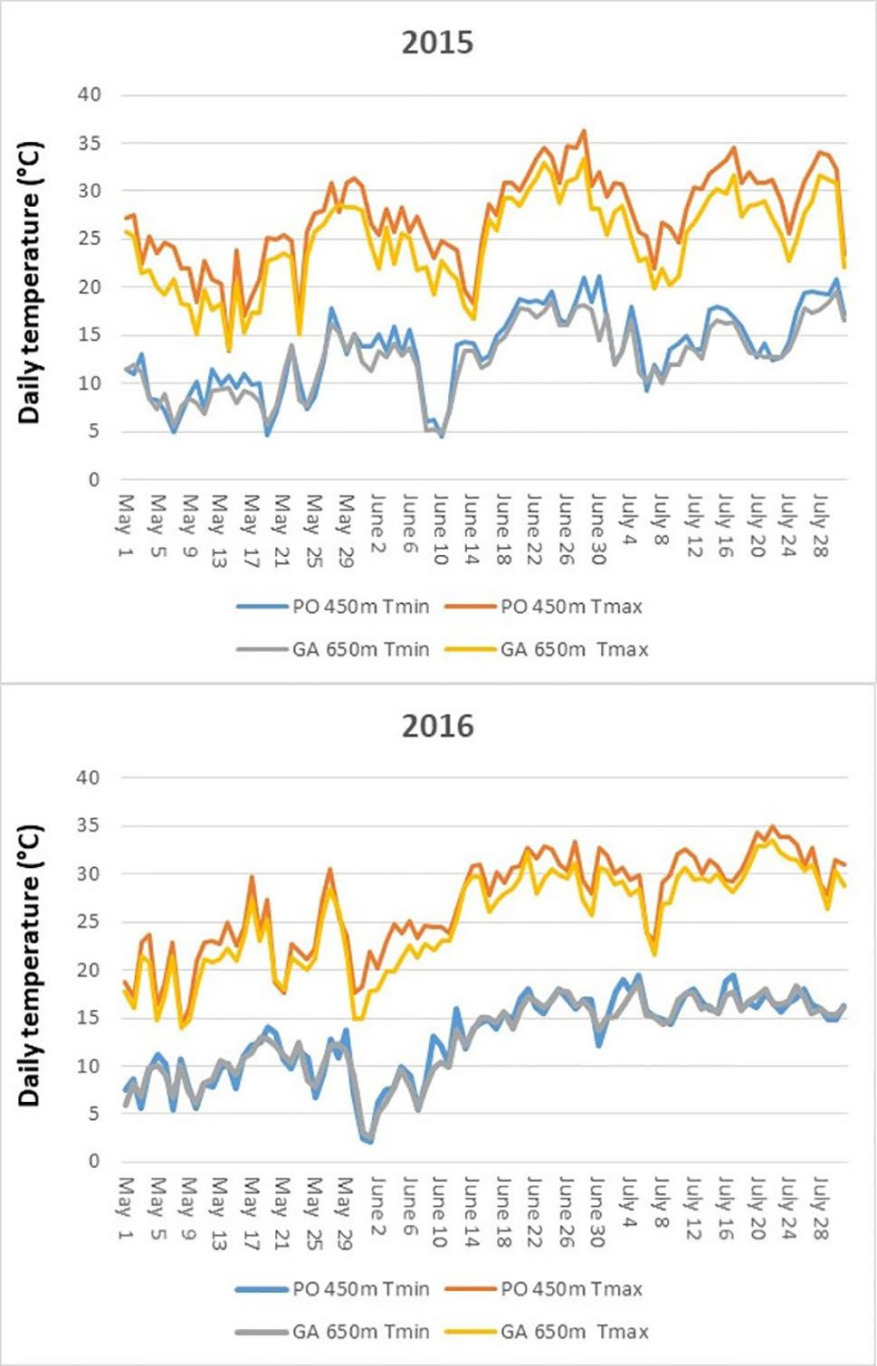
Maximum daily temperatures and minimum daily temperatures recorded along in the two growing locations of Postalesio (450 m) and Gaggio (650 m) during berry development and ripening (May–July) in 2015 and 2016.

The course of daily maximum and minimum temperatures for the period May–July of both seasons for each site is reported in **Figure 2**. Minimum temperatures were very close in the two sites with an average difference of 0.8°C in 2015 and 0.2°C in 2016. Maximum temperatures were constantly higher in Postalesio, with +2.6°C on average in 2015 and +1.6°C in 2016 over the period May–July. As a consequence, daily thermal excursion was higher in Postalesio, with an average excursion in the May–July of 13.8°C in 2015 and 13.9°C in 2016, while in Gaggio, it was 12.1°C and 12.4°C, respectively.

Considering thermal resources useful for blueberry growth, growing degree days with 0°C base (White et al., 2012) were calculated for the May–July period: in agreement with temperature courses, Postalesio reached higher values of 1877 in 2015 and 1833 in 2016 GDD, while Gaggio 1718 and 1749 GDD.

Regarding solar radiation, PPAR, calculated by means of the topographic tools of SAGA GIS, reached in Gaggio 3,544 MJ/m^2^, while it reached 3,109 MJ/m^2^ in Postalesio.

Since the two sites are very close from one to the other and on the same side of the valley, we can assume similar patterns in the cloudiness and argue that the proportion of real PAR between the two sites is the same as for Potential PAR.

The monthly cumulated Potential PAR showed constantly higher availability of radiation (+17% on average) in Gaggio (421, 466, 498 MJ m^−2^ in May, July, and June, respectively) over Postalesio (362, 398, 423 MJ m^−2^ in the same months).

Similar results were obtained simulating real PAR on the base of maximum and minimum daily temperatures by means of the Hargreaves model (Allen et al., 1998) (**Supplementary Figure S2**); considering the experimental time span May–July, Gaggio showed on average +15% of PAR in 2015 and +14% in 2016 compared to Postalesio.

### Total Phenolics, Total Flavonoids, and Total Anthocyanins

**Figure 3:**
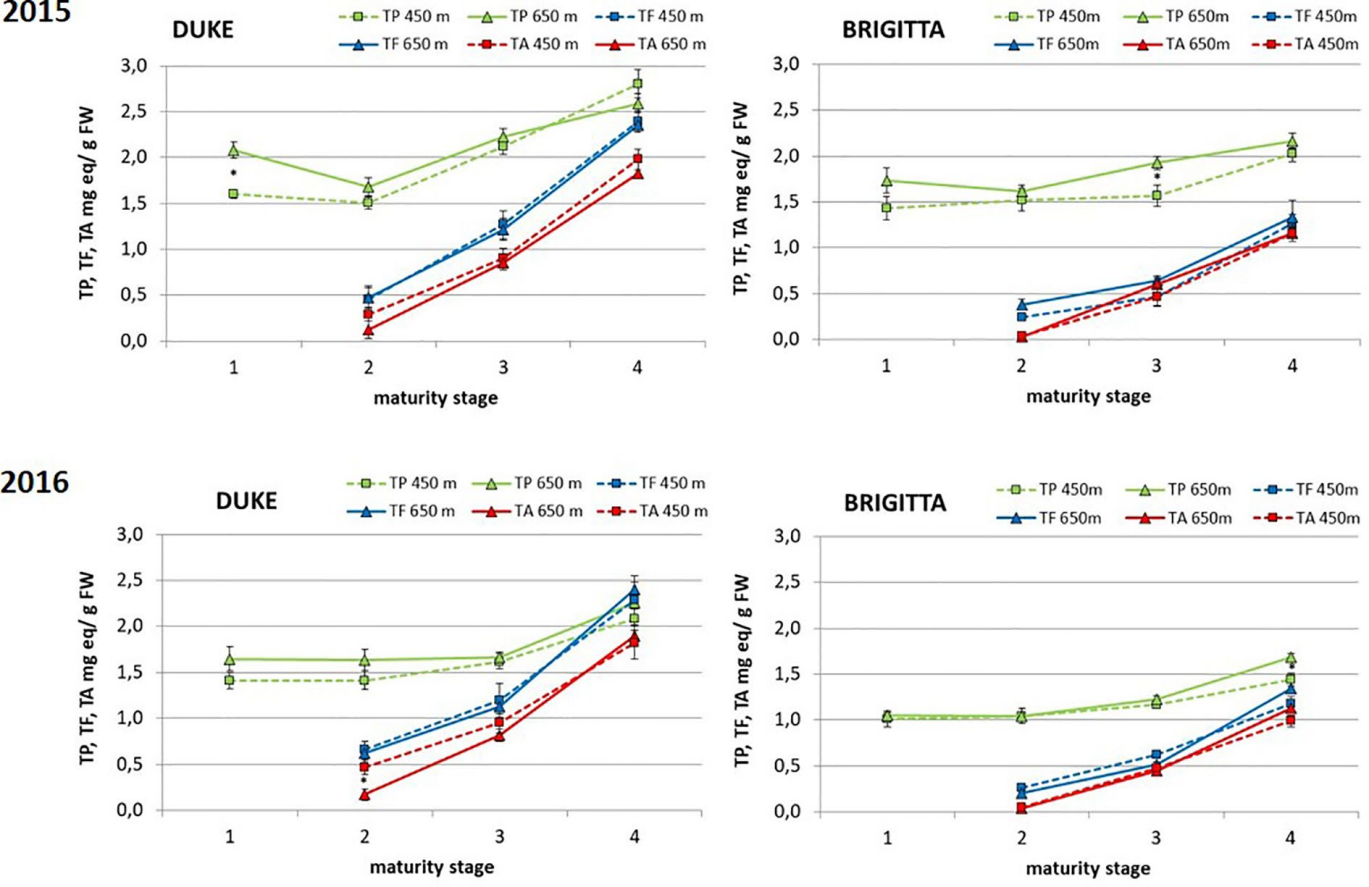
Total phenols (expressed as mg catechin equivalents/g FW), total flavonoid (expressed as mg catechin equivalents/g FW) and total anthocyanins (expressed as mg malvidin 3-glucoside equivalents/g FW) (mg/100 g FW) in blueberries cv. “Duke” and “Brigitta” in 2015 and 2016. Values are means of six replicates. Asterisks indicate statistical differences between altitudes (P ≤ 0.05).

“Duke” exhibited in both years higher amounts of total phenols than “Brigitta” throughout development and ripening (**Figure 3**). In both cultivars, the levels did not change markedly in the first two classes and then slightly increased due to the accumulation of anthocyanins starting from ripening class II. During the two growing seasons, ripe berries of “Duke,” compared to berries of class II, presented an average increase in total phenol concentrations of 66% at the lower altitude and an average increase of 46% at the higher growing site. Conversely, in “Brigitta,” total phenol accumulation, comparing class II and class IV fruits, increased averagely by 35% at the lower site and by 47% at the higher site. There was no clear effect of different altitudes on this parameter, although in 2015, “Duke” class I and “Brigitta” class III and in 2016 “Brigitta” class IV polyphenol contents were greater at higher altitude.

Remarkably, the pattern of accumulation of polyphenols did not change between years, even if the amounts were significantly higher in 2015 respect to 2016, both for “Duke” and for “Brigitta.”

Similar trends were observed for total flavonoids and total anthocyanins, analyzed in berries starting from class II (turning stage) (**Figure 3**). The accumulation of these two groups of phenols increased progressively throughout ripening, determining significant differences among the three classes of both blueberry cultivars. During the two subsequent years, anthocyanin concentrations in “Duke” berries increased during ripening averagely by 432% and by 1,192% at the lower and at the higher growing sites, respectively. In “Brigitta” berries, anthocyanin accumulation was more pronounced than in “Duke,” as fruit of “Brigitta” at stage II presented lower pigment levels than fruit of “Duke.” In “Brigitta” fruits, the anthocyanin concentrations augmented 25-fold during maturation at lower and 34-fold at higher altitudes. As shown in **Figure 3**, total flavonoids and total anthocyanins were always higher in “Duke” compared to “Brigitta.” As for polyphenol content, altitude did not affect total flavonoid and total anthocyanin contents and did not alter their pattern of accumulation.

### Anthocyanin Profile

Anthocyanin profiles of “Duke” and “Brigitta” were systematically analyzed over the ripening process starting from the beginning of fruit pigment accumulation at stage II. The anthocyanins identified in both cultivars were monoarabinosides (ara), monogalactosides (gal) and monoglucosides (glu) of delphinidins (Dp), cyanidins (Cy), petunidins (Pt), peonidins (Pn), and malvidins (Mv) (**Supplementary Figure S3**; **Supplementary Table S1**). The acetylated form Pt-acetyl-galactoside was also detected in both varieties. In “Brigitta” berries, the predominant anthocyanidins were Dp and Mv. In ripe fruits of “Duke,” the main anthocyanidin was Mv followed by Dp and Pt. As in “Brigitta,” Cy and Pn were in minor proportion. The anthocyanidins identified were in both varieties conjugated prevalently with gal and secondarily with ara. Only Mv was detected as glu.

**Figure 4:**
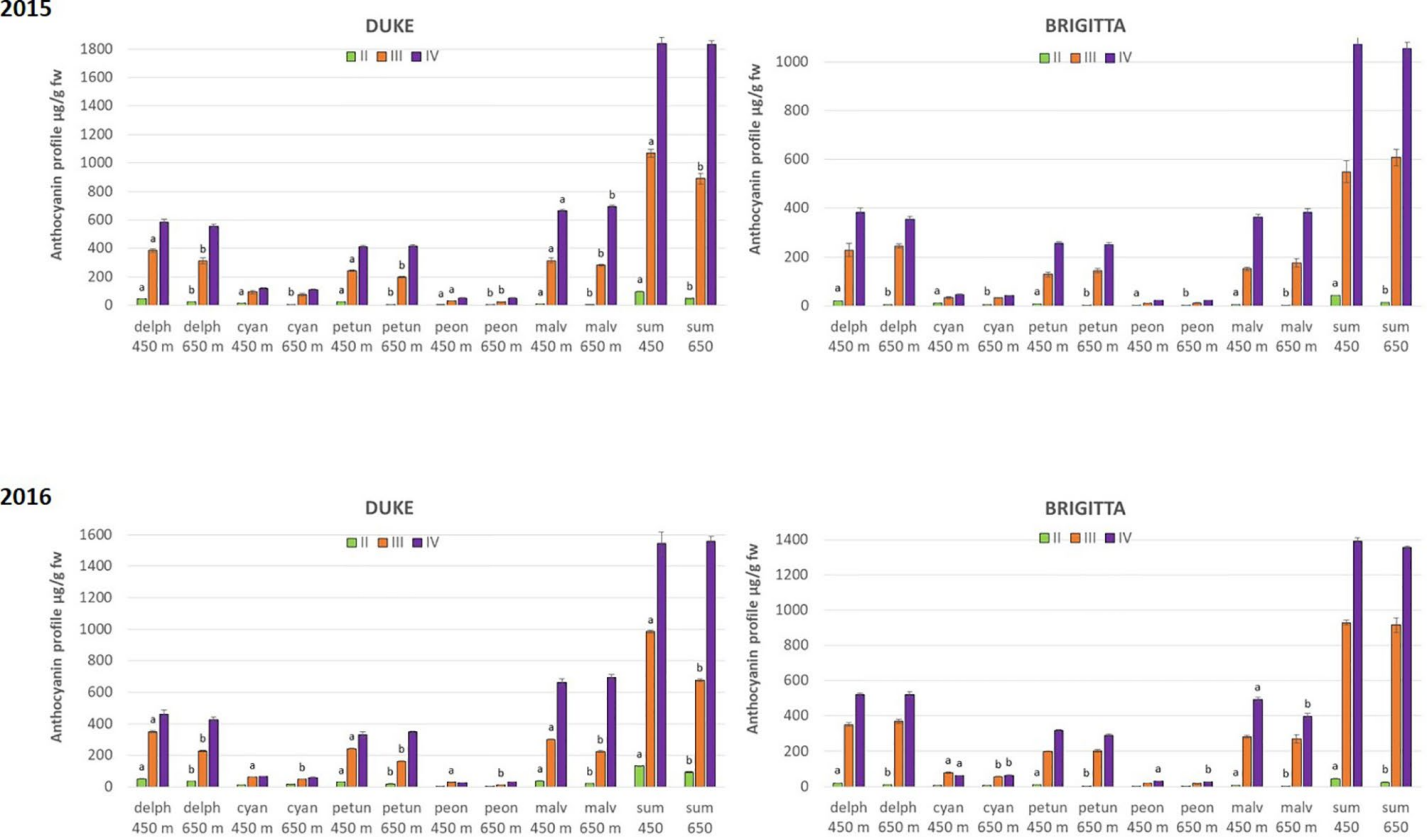
Anthocyanin accumulation (µg/g FW) in blueberries cv. “Duke” and “Brigitta” in 2015 and 2016. Values are means of six replicates. Different letters indicate statistical differences between altitudes for each ripening class (P ≤ 0.05).

The total amount of anthocyanins, calculated as the sum of all the individual anthocyanins resulting by the chromatographic profiles, showed significant differences among all the classes of ripening, confirming the results obtained by the spectrophotometrical analysis (**Figure 4**).

Importantly, the chromatographic analysis revealed differences also between growth locations at particular berry developmental stages. During berry development, anthocyanin amounts were higher in berries grown at lower altitudes. “Duke” berries of classes II and III accumulated more anthocyanins at 400 m in both years, whereas in “Brigitta,” higher levels of pigments were observed at 400 m in class II in both years. Berries of “Duke” and “Brigitta” class IV (full-ripening stage), however, did not differ in total anthocyanin amounts in any growing season. Remarkably, Dp and Pt glycosides reached significant higher amounts in fruits of class II at the lower growing location, regardless of the cultivar and growing season.

**Figure 5:**
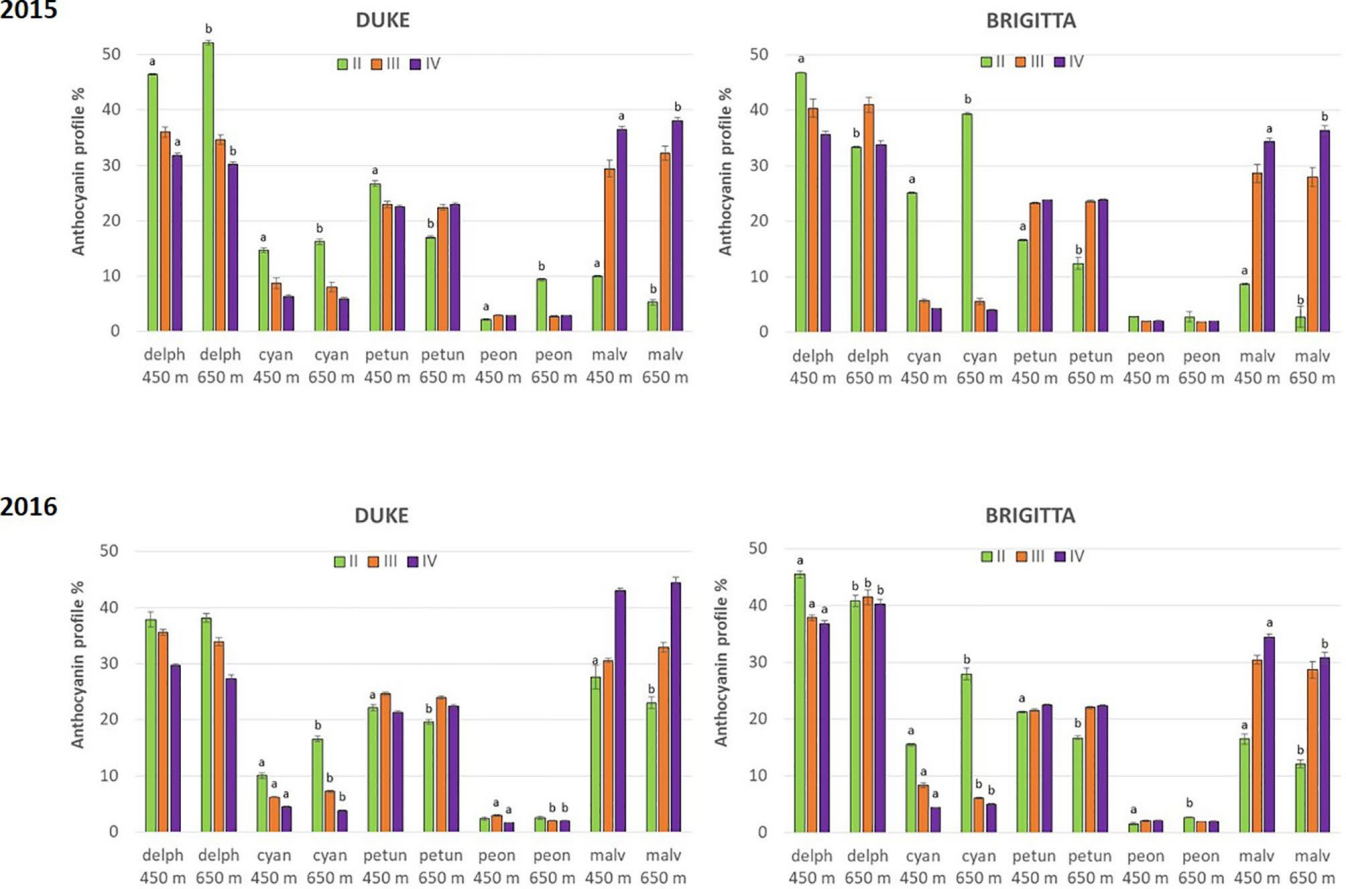
Relative proportion of individual anthocyanins (%) in blueberries cv. “Duke” and “Brigitta” in 2015 and 2016. Values are means of six replicates. Different letters indicate statistical differences between altitudes for each ripening class (P ≤ 0.05).

**Figure 6:**
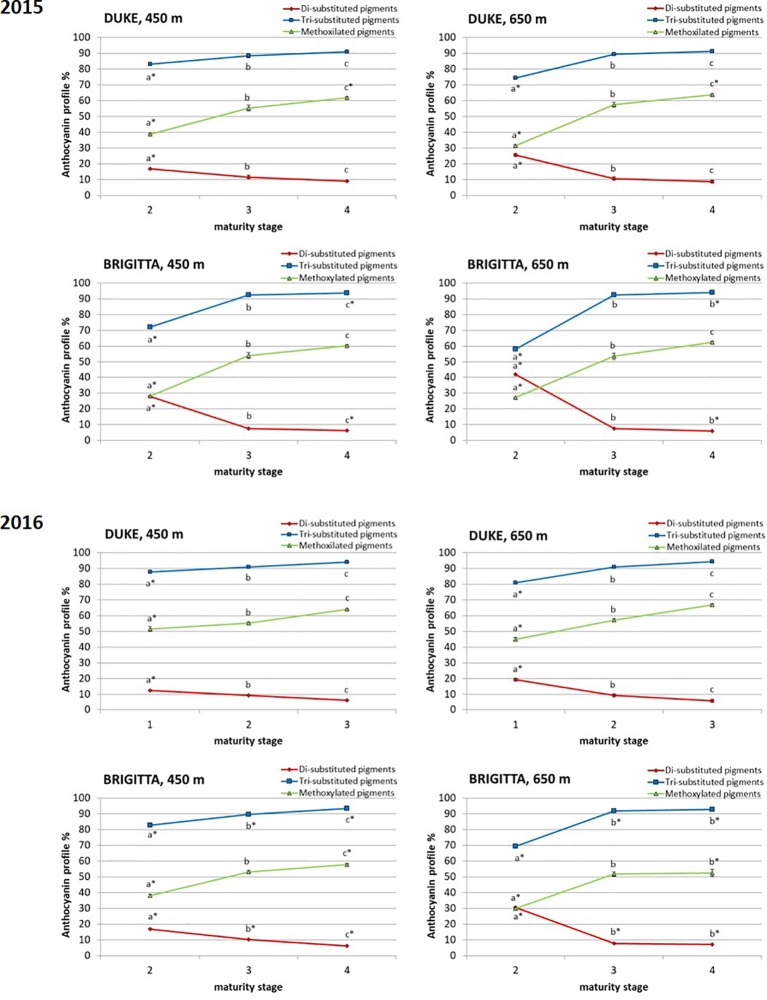
Relative proportion of di-substituted anthocyanins, tri-substituted anthocyanins, and methoxilated anthocyanins (%) in blueberries cv. “Duke” and “Brigitta” in 2015 and 2016. Values are means of six replicates. Asterisks indicate statistical differences between altitudes (P ≤ 0.05). Different letters indicate statistical differences among ripening classes of each cultivar (P ≤ 0.05).

In addition to absolute concentrations, it is useful to express anthocyanin composition in relative terms (**Figure 5**). Considering the relative proportion of the individual anthocyanins during blueberry ripening, a shift of anthocyanin biosynthesis from cyanidin-type, di-substituted molecular structures (Cy, Pn) toward delphinidin-based, tri-substituted pigments (Dp, Pt, Mv) was evident. At color appearance, di-substituted anthocyanin relative proportions were highest. During ripening, however, a progressive increase in the proportion of tri-substituted and methoxylated (Pn, Pt, Mv) pigments was observed (**Figure 6**). The proportions of tri-substituted and methoxylated anthocyanins were always lower at higher altitude at the early stage of ripening (class II). Conversely, Cy and Pn glycosides reached higher proportions in berries belonging to class II grown at the higher sites, in both cultivars and years.

**Figure 7:**
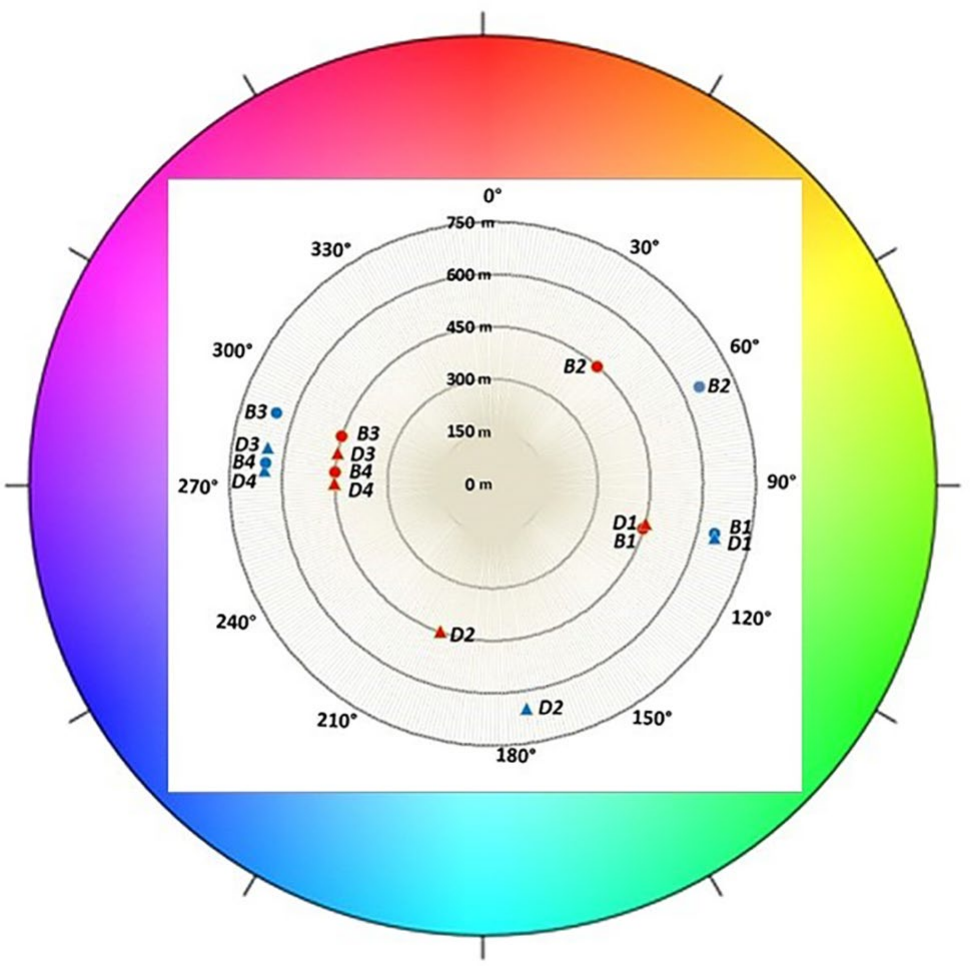
Color expressed as hue angle (degrees) recorded in different classes of ripening (1, 2, 3, 4) of blueberries cv. “Duke” (D) and “Brigitta” (B) grown at two different altitudes. Polar plot: 0° and 360° indicate red, 90° indicates yellow, 180° indicates green, and 270° indicates blue. Values are means of 30 replicates recorded in 2015.

**Figure 8:**
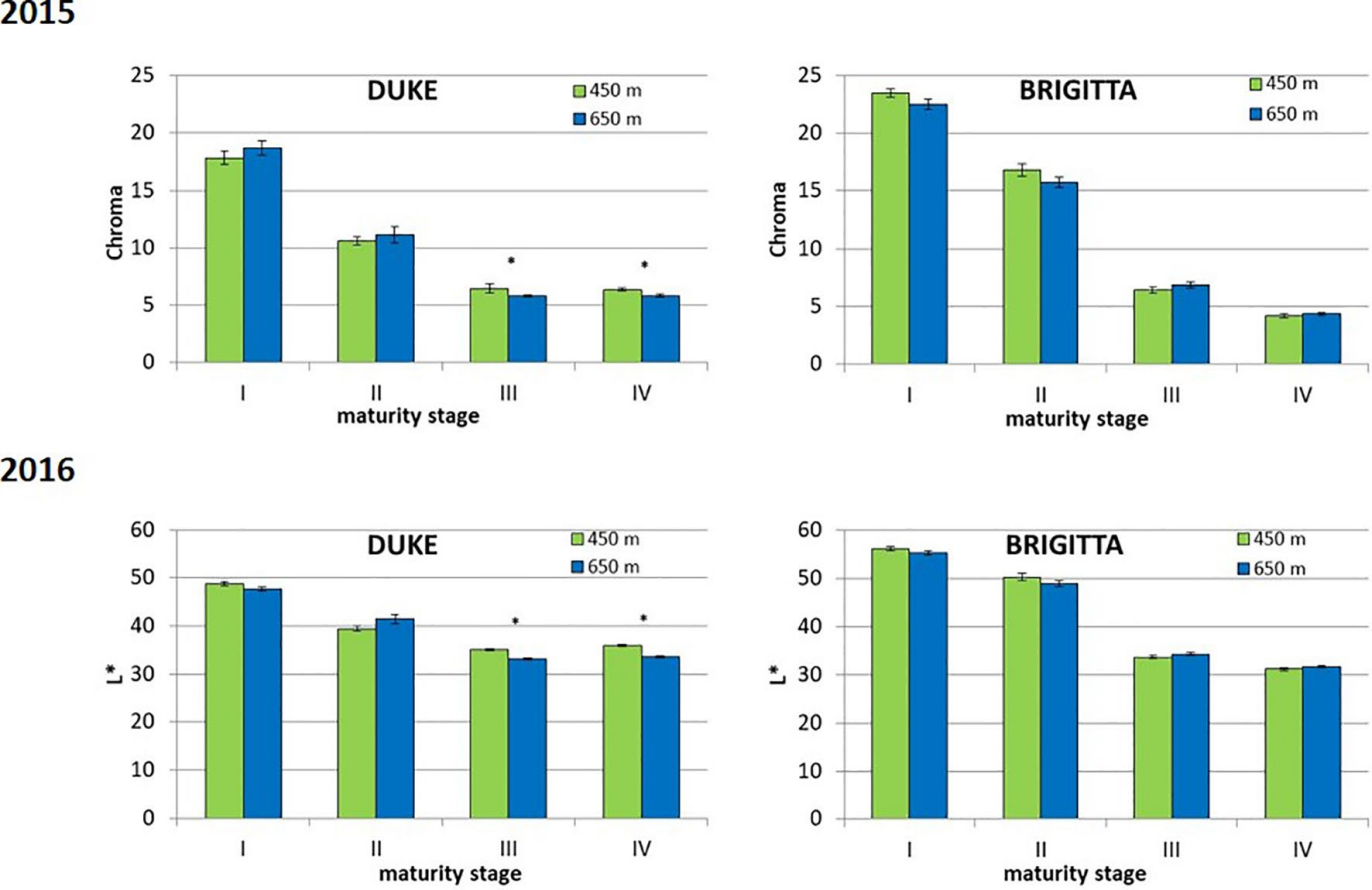
Chroma (top) and L* (bottom) recorded in different classes of ripening of blueberries cv. “Duke” and “Brigitta” grown at two different altitudes. Asterisks indicate statistical differences between altitudes (P ≤ 0.05). Values are means of 30 replicates recorded in 2015.

In the first growing season, fruit skin color was determined (**Figure 7**). No distinction was perceived between unripe fruits (class I) of both cultivars, which displayed a green color. Berries belonging to class II, associated to the initial appearance of pigment accumulation, showed genotype-specific differences. “Brigitta” skin color shifted toward a reddish hue (average hue value moving toward lower values) whereas “Duke” fruit tended to a bluish coloring (average hue value moving toward higher values). This is in line with the ratio of red-/cyanidin- and blue-/delphinidin-based anthocyanins found in the two cultivars at the breaker stage. Moreover, fruit skin hue of blueberries grown at the lower altitude location showed more pigmentation with decreased values for “Brigitta” and increased values for “Duke.” Fruit belonging to classes III and IV of both cultivars showed a shift from a violet (290°) to a blue color (210°). During blueberry ripening, chroma values progressively decreased as berry skin colors became duller (**Figure 8**). On the other hand, L* values also decreased at the same time (**Figure 8**), due to the peel becoming increasingly pigmented and darker in the course of maturation.

## Discussion

### Environmental Factors

The availability of environmental resources strongly affects quality and quantity of plant production (Larcher, 1995; van Keulen and Wolf, 1986), with a primary role played by air temperature, solar radiation, and precipitation. Air temperature is the direct result of the energy balance driven by solar radiation and is influenced by topography, soil physical and chemical characteristics, soil management and water content, canopy shape, and management.

Similar topographic features, soil characters and same management (training system, cultural practices, and water supply) characterize the two sites object of this study. Consequently, we can assume that most of the environmental variability can be associated with the different availability of solar radiation by the specific courses of air temperature, both strongly related to altitude.

The current study focuses on the thermal course of the two sites, where higher availability in thermal resources determined quicker phenological development and affected ripening processes and anthocyanin accumulation. A complementary approach focuses on the relevant role played by solar radiation, taking into consideration other flavonoids such as flavonols. In fact, flavonol synthesis is upregulated by solar radiation, which shapes the flavonol profile, and especially the proportion of kaempferol, over time (Martínez-Lüscher et al., 2019). Additionally, the quality of solar radiation and its relationship with elevation and other geographic factors (Brillante et al., 2017; Martínez-Lüscher et al., 2019; Zoratti et al., 2015a) should be also investigated in order to assess the effects on the dynamics of metabolites—for instance, the relationship between variations of UV with elevation (Blumthaler et al., 1997, Diffey, 1991).

In the same environment of the present study and on grapevine, Failla et al. (2004) analyzed the spatial distribution of solar radiation and the effects on vine phenology and grape ripening. They concluded that different factors cooperated in the phenological timing of plants and in their ripening dynamics, with temperature (decreasing with altitude) and PPAR (increasing with altitude in the viticultural belt) as predictors for the considered variables.

### Total Phenolics, Total Flavonoids, and Total Anthocyanins

Phenolic compounds including flavonoids (i.e., flavanols, anthocyanins and flavonols) and phenolic acids are considered as nonnutrient biologically active compounds and concur in determining the antioxidant capacity of fruits. In blueberry, antioxidant activity correlates well with total phenolic content and anthocyanin content (Prior et al., 1998; Ehlenfeldt and Prior, 2001).

The increasing trends of “Duke” and “Brigitta” total phenols in 2015 and 2016 were partially different from the accumulation patterns reported for other varieties in Germany, which showed a decrease or steady level during color break and ripening (Castrejón et al., 2008). In Nova Scotia, blueberries displayed no changes or increasing levels during ripening and a decrease at full ripeness (Kalt et al., 2003). Differences of our results in comparison to those obtained in these studies may arise due to genotypic variation, different cultivation, and climate conditions and to different extraction solvents and analytical methods used. Moreover, contribution to blueberry total phenolic content is mainly associated to the phenolic acid group of hydroxycinnamic acids derivatives and to the flavonoid subclasses of flavanols (and their polymers proanthocyanidins), flavonols and anthocyanins. Flavonols, proanthocyanidins, and hydroxycinnamic acids and the corresponding transcripts are most concentrated in young fruits. By contrast, the levels of these compounds markedly decrease at later stages of berry development, the accumulation of anthocyanins begins at the onset of ripening, and they are the major flavonoids in the ripe berry (Jaakola et al., 2002; Castrejón et al., 2008; Zifkin et al., 2012; Vvedenskaya and Vorsa, 2004). Polyphenolic content therefore integrates all the different accumulation patterns of these classes of compounds that may vary among cultivars (Ribera et al., 2010; Guofang et al., 2019). Comparison of six highbush blueberry cultivars and *Vaccinium angustifolium* showed relative low amounts of chlorogenic acid and proanthocyanidins and high contents of anthocyanidins in “Brigitta” berries, suggesting a relative high contribution of the pigment class of phenols to the total phenolic content. Starting from turning stage (class II), “Duke” and “Brigitta” steadily accumulated flavonoids and anthocyanins. These results are in line with those of Ribera et al. (2010) who reported total anthocyanin concentrations for “Brigitta” ranging from 3.79 to 190 mg cyanidin-3-glucoside 100 g^−1^ FW and with comparable trends in rabbiteye blueberry (Guofang et al., 2019), in lowbush blueberry (Gibson et al., 2013), and bilberry (Jaakola et al., 2002).

Beside the strongly consistent developmental pattern and the genotypic effect, growth altitude did not apparently affect the total amounts of flavonoids and anthocyanins, nor did it the harvest season.

### Anthocyanin Profile

The anthocyanin profile of ripe berries of “Brigitta” was similar to the profiles reported in other studies (Rodriguez-Mateos et al., 2012; Zoratti et al., 2015b); Dp and Mv were the dominant forms, followed by Pt and, in lowest proportion, Cy and Pn. In ripe fruits of “Duke,” the main anthocyanidin was Mv; Cy and Pn were present in minor proportion. Similar results were reported in a study on blueberries grown in China (Li et al., 2016).

Anthocyanin accumulation begins at the onset of maturation and proceeds until full ripening. The flavonoid pathway has been intensively studied in cultivated and wild berry species, such as blueberries and bilberries, and the main structural genes have been isolated from highbush blueberry (Zifkin et al., 2012) and bilberry (Jaakola et al., 2002). The researches have indicated the increase in transcription levels particularly of chalcone synthase (*CHS*), dihydroflavonol 4-reductase (*DFR*), anthocyanidin synthase (*ANS*), and UDP-glucose flavonoid 3-*O*-glucosyltransferase (*UFGT*) at the ripening stage leading to anthocyanin accumulation. In addition, several transcription factors that play a role as key regulators of the flavonoid pathway have been identified (Falcone Ferreyra et al., 2012).

In our study, anthocyanin concentrations during berry development were higher in berries grown at lower altitudes. Remarkably, berries at turning stage accumulated more anthocyanins at the lower altitudes in both years. Berries of “Duke” and “Brigitta” class IV (full-ripening stage), however, did not differ in total anthocyanin amount in any growing season. The results are partially in accordance with the data on ripe bilberry grown at different altitudes in the eastern Alps of Austria (Rieger et al., 2008). The influence of altitudinal variation on the anthocyanin content of bilberry fruits was evident in both years, with a decrease moving from 800 to 1,200 m; in particular, all the individual anthocyanin levels were lower at higher altitude. Conversely, fruits grown at the highest altitude of 1,500 m showed only few differences compared to those at 1,200 m. Different results were reported by Zoratti et al. (2015b) on blueberry cv. “Brigitta” and bilberry grown in the eastern Alps of Italy. Bilberry revealed a progressive increase in anthocyanin accumulation along the altitudinal gradient of about 650 m. On the other hand, the effect of altitude increase on “Brigitta” was not clear, and contradictory data were obtained in the two growing years. These seasonal differences in anthocyanin pattern of accumulation were considered linked to environmental effects and to the markedly different temperatures recorded in some locations during the development and ripening of the fruits in the two growing years. In our study, temperatures at the lower altitude appeared to affect positively anthocyanin accumulation, mainly in the early ripening cv. “Duke.” Differences in maximum daily temperatures between the two locations were about 2.6°C in the first half of July 2015 (average maximum temperatures of 24.7°C in Berbenno *vs*. 22.1°C in Gaggio) and about 2.2°C in the second half (average maximum temperatures of 32.0°C in Berbenno *vs*. 29.8°C in Gaggio). In the subsequent year, the temperature flow recorded in the same period did not differ substantially (the data recorded were 25.1°C in Berbenno *vs*. 23.0°C in Gaggio in the first half of July 2016 and 30.9°C and 28.9°C, respectively, in the second half). Optimum temperatures for blueberry fruit set and ripening are 20–26°C during the day and 16°C at night, although genetic variation has been documented (Lobos and Hancock, 2015). Therefore, “Duke” appeared to take advantage of the higher nighttime and daytime temperatures during color break and ripening by accumulating more anthocyanins within the different developmental stages. In “Brigitta,” which ripens later, the positive effect of the higher temperature at the lower location was visible only at the onset of ripening, when the berry started pigmentation (class II). After this stage, the patterns of pigment accumulation were similar at both altitudes, suggesting that the environmental conditions allowed the kinetic of pigment deposition coordinated to the ripening process to proceed faster in berries grown at the higher location. Pattern of phenol accumulation and related gene expression observed in blueberry, bilberry, strawberry, and grape (Zifkin et al., 2012; Jaakola et al., 2002; Carbone et al., 2009; Castellarin et al., 2007a) highlights the coordination of phenol biosynthesis with the developmental stages of the fruit. Therefore, environmental factors that alter the rate of berry development may indirectly affect metabolite, e.g., anthocyanin accumulation, and environmental effects impacting particular metabolites would be time-dependent.

Zoratti et al. (2015b) reported as optimal temperature conditions for “Brigitta,” high maximum daily temperatures (as high as 33.5°C) during the pink stage, followed by lower temperatures fluctuating around an average of 26°C until ripening, relatively higher than optimum temperatures of literature. Ideal conditions for anthocyanin accumulation are nowadays not known, and it has not yet been established whether anthocyanin accumulation is due to an integrated response to temperature as expressed by the concept of thermal time, to specific threshold temperatures, or to a duration of exposure to critical temperatures. Likewise, the critical stage of development for a response to temperature has not been characterized. In grape, *in vitro* study indicated that a key time point might be the 2 weeks following the first skin color appearance (Yamane et al., 2006).

In our study, the temperature conditions at different altitudes had significant but temporary effects on anthocyanin accumulation. In both blueberry cultivars, berries appeared to compensate for these initial temperature effects. Higher temperatures during day and night led to an initial difference in anthocyanin concentration, but subsequently, berries at the final stage of ripening counterbalanced the gap through accumulating pigments at a more rapid rate, resulting in equal levels recorded at stage IV. Similar compensation mechanisms could be associated to observations reported in grape by Gouthu et al. (2014) which showed that, in a cluster, during the last phase of fruit maturation, the ripening rate of under-ripe berries is higher than that determined in the ripest berries to reach a synchronized development. Also, Corso et al. (2016) reported that grapevine rootstocks, characterized by a different effect on the rate of ripening of “Cabernet Sauvignon” berries, although determining different timings at the beginning of fruit development, (e.g., an earlier onset of ripening in the graft combination CS/M4 compared to CS/1103P), did not affect skin colorimetric parameters of berries at full-ripening stage. These results suggest an acceleration of ripening induced by 1103P rootstock at last stages of maturation and the need of the ripening transcriptional program to be completed in a genetically defined temporal window, independently by exogenous factors affecting the early phases of berry ripening initiation.

Climate differences associated to different altitudes affected accumulation of individual anthocyanins in “Duke” and “Brigitta” (**Figure 4**). In both years, the tri-substituted anthocyanins Dp, Pt, and Mv reached higher levels at the lower location, characterized by higher temperatures, during the early stages of fruit ripening (stages II and III of “Duke” and stage II of “Brigitta”). An opposite trend has been reported in bilberry, which increased accumulation of tri-substituted anthocyanins with increasing altitude (Zoratti et al., 2015b) and in grape “Pinot noir” grown at high daytime temperatures, which reduced the levels of Dp 3-glucoside, Pt 3-glucoside, and Mv 3-glucoside in the berry skin (Mori et al., 2007). Tarara et al. (2008) showed in a complex study on “Merlot” grapes, in which temperature-control regimens were dynamic paralleling the diurnal temperature fluctuation in the vineyard that, with increasing berry temperature, total concentrations of Dp, Cy, Pt, and Pn-based anthocyanins decreased, whereas the total concentration of Mv-based anthocyanins was unaffected. The temperatures, particularly daytime temperatures during ripening, appeared to be an environmental determinant of anthocyanin accumulation in blueberry under the field conditions encountered in this study. Moreover, the higher temperatures encountered at the lower altitude location may not have exceeded optimum for maximum anthocyanin accumulation, resulting in a quicker berry developmental pattern and in higher anthocyanin concentrations in the early phases of ripening, compared to the higher location.

Anthocyanin production is controlled by several transcription factors that affect the ratio of di-/trihydroxilated anthocyanins through trans-regulation of flavonoid 3´-hydroxylase (*F3’H*) and flavonoid 3’5’-hydroxylase (*F3’5’H*) gene expression. The biosynthesis of di-substituted anthocyanins is promoted by the enzyme F3’H activity, which is responsible for the hydroxylation of the precursor dihydrokaempferol at position 3´ of the B-ring and promotes Cy and Pn accumulations. F3’5’H activity drives the synthesis of tri-substituted anthocyanins, being responsible for the hydroxylation at the 3’,5’-positions of the B-ring, that results in the production of Dp and the methylated derivatives Mv and Pt. Increase in flavonoid 3’5’-hydroxylase activity also diverts the biosynthesis from the Cy and Pn branch toward the branch resulting in Dp, Mv, and Pt as final anthocyanin structures. In blueberry, *F3’5’H* is weakly expressed during the earliest ripening stages and increases during the late ripening stages, closely paralleling the accumulation of tri-substituted anthocyanins (Zifkin et al., 2012).

Early in the ripening process of “Duke” and “Brigitta” blueberries, Cy-type anthocyanins were abundant, likely accounting for the red blush to parts of the green berry. As the fruit ripened and the exocarp color changed from mostly green and reddish to partially pink, blue-purple Dp-type anthocyanins began to accumulate. The appearance of the trihydroxylated anthocyanidin Dp and its derivatives Mv and Pt is coordinated with the abundance of *VcF3’5’H* transcripts at developmental stage coincident with the onset of ripening (Zifkin et al., 2012). Similar results were observed in grape, where the correlation between transcript profiles and the kinetics of accumulation of red-/cyanidin- and blue-/delphinidin-based anthocyanins indicated that *VvF3’H* and *VvF3’5’H* expressions were consistent with the chromatic evolution of ripening bunches (Castellarin et al., 2006). In the present study, developmental regulation based on genotypic information appeared to be more relevant and environmental factors related to different altitudes had only fine-tuning influence. The present results are comparable with studies conducted on bilberry (Jaakola et al., 2004) and on strawberry (Carbone et al., 2009), in which anthocyanin accumulation showed to be under strong developmental control. Moreover, light seems to play a secondary, fine-tuning role on the accumulation of flavonoids in different *Vaccinium* species. Many of the wild *Vaccinium* berries, like bilberries, grow in shaded habitats and do not require high light for induction of anthocyanin biogenesis. Blueberries are also shade-adapted species, although they seem to require higher solar exposure for normal ripening and anthocyanin accumulation (Zoratti et al., 2015a). In fact, regarding anthocyanin production, fruits can be classified into those that have anthocyanins in both their skin and flesh, those that have anthocyanins only in their skin, and those that accumulate anthocyanins in their skin only as response to a light stimulus. In the first two classes, developmental regulation has a major role in anthocyanin biosynthesis, whereas in the third, anthocyanin biosynthesis is more under environmental control (Jaakola, 2013). There is strong evidence that blueberry and bilberry belong to the first group. Findings reported by Karagiannis et al. (2016) on the regulation of peach skin quality traits by altitude, conversely, suggested that, in this case, environmental factors had a more important role in skin anthocyanin accumulation and higher altitude favored peach pigment biosynthesis, as already proposed in apple (Lin-Wang et al., 2011).

Our results indicate that, during bilberry ripening, when the relative proportion of individual anthocyanins is considered, the shift of biosynthesis from cyanidin-type, di-substituted molecular structures toward delphinidin-based, tri-substituted pigments were evident. De Lorenzis et al. (2016) reported similar trends in “Corvina” grape. Grapes subjected to different water stress conditions showed an analogous behavior, with an altered ripening process and subsequent anthocyanin profile (Brillante et al., 2017). In fact, skin total amount of anthocyanins was negatively affected by severe water stress; in particular, the dihydroxylated forms were more affected than trihydroxylated. At the same time, a pronounced water stress reduced net carbon assimilation, but not TSS, which, on the contrary, increased. Since the berry mass was not significantly affected, the authors suggested that higher water stress accelerated ripening. Moreover, the observed reduction of TA could be related to water stress, but could also be linked to a more advanced ripening in the severely stressed condition. Therefore, the increase in the tri-/dihydroxylated ratio could be related to a stressful factor, as the anthocyanin biosynthesis appeared to be shifted toward the production of more complex and stable molecules, and to an acceleration of the ripening process.

In the present study, blueberry plants at both altitudes were well watered throughout the developmental stages, and no stresses due to water deficit or other soil properties were expected. Moreover, tri-substituted and methoxilated anthocyanin proportions were always lower at higher altitude at the early stage of berry ripening and were likely associated with a slower progression of the ripening process. The developmentally regulated proportion changes between di- and tri-substituted anthocyanins occurred earlier at the lower altitude location characterized by higher temperatures, where blueberries initially ripened more quickly. These differences however diminished along the ripening process and suggest that berries grown at higher altitude compensated for this initial disparity through accumulating tri-substituted pigments at a much more rapid rate as the temperature became higher, closer to the optimum. In “Brigitta,” this was more evident compared to “Duke,” and the increase in the proportion of tri-substituted anthocyanins between class II fruit and class III fruit was more steep. This compensatory mechanism suggests a feedback response resulting in part from the coordinated regulation of flavonoid pathway genes, mainly driven by developmental and genetic cues and by specific environmental effects (Cohen et al., 2012). Moreover, a different response in anthocyanin accumulation between “Brigitta” and “Duke” was also reported following methyl jasmonate treatment (Cocetta et al., 2015).

Fruit skin color evaluated in the first growing season was in line with the ratio of red-/cyanidin- and blue-/delphinidin-based anthocyanins found in the two cultivars at the breaker stage. Fruit skin hue of blueberries grown at the lower altitude location showed more pigmentation with decreased values for “Brigitta” and increased values for “Duke.” Fruit belonging to classes III and IV of both cultivars showed a shift from a violet (290°) to a blue color (210°), a trend associated with the progression in the ripening process.

The observed progressive decline in the chroma values may be an effect of epicuticular waxes deposition during berry development (Saftner et al., 2008; Konarska, 2015). Indeed, cuticular wax load (fruit bloom) increased in blueberry during the fruit development, leading to a thick cuticle at maturity (Chu et al., 2018; Trivedi et al., 2019) and resulting in less vivid skin color. On the other hand, L* values also decreased at the same time and reflected anthocyanin accumulation rather than fruit bloom, in accordance with the data reported in other blueberry cultivars (Chung et al., 2016; Matiacevich et al., 2013).

## Conclusions

The present study suggests that anthocyanin accumulation is strongly regulated by development and genotype. The environmental effect exerted by temperature, when not far exceeding optimum, on anthocyanin concentration and composition in blueberry fruit is mainly associated to the pattern of ripening progression. Furthermore, this fine-tuning influence may only be temporary, and with increasing temperatures, differences in anthocyanin levels may disappear due to compensatory mechanisms in anthocyanin accumulation.

## Data Availability

All datasets generated for this study are included in the manuscript and the supplementary files.

## Author Contributions

AS and IM developed the concept of the paper. AS wrote the paper, performed spectrophotometric analyses and colorimetric determinations, and together with CG performed the analysis of anthocyanin profile by HPLC and MS. GC provided the environmental data. All authors discussed and commented on the manuscript.

## Funding

Funds were received for open access publication fees from Università degli Studi di Milano, Attività Base di Ricerca (FFABR)—2017.

## Conflict of Interest Statement

The authors declare that the research was conducted in the absence of any commercial or financial relationships that could be construed as a potential conflict of interest.
