# Supplementary material for: Variation of Anthocyanin Content and Profile Throughout Fruit Development and Ripening of Highbush Blueberry Cultivars Grown at Two Different Altitudes
Source: Front Plant Sci. 2019 Sep 4;10:1045. doi: 10.3389/fpls.2019.01045 (PMC6737079; doi:10.3389/fpls.2019.01045)
Supplement: Supplementary file 1 [file DataSheet_1.pdf]

SUPPLEMENTARY TABLE S1. UHPLC-HR-MS data for anthocyanin identification.

Peak	Anthocyanin	$[M]^+$	Fragment ion$[M]^+$
1	D-gal	465.1026	303.0499
2	D-glc	465.1026	303.0500
3	Cy-gal	449.1079	287.0548
4	D-ara	435.0920	303.0500
5	Cy-glc	449.1079	287.0548
6	Cy-ara	419.1000	287.0548
7	Pet-gal	479.1180	317.0655
8	Pet-glc	479.1180	317.0655
9	Peo-gal	463.1232	301.0705
10	Pet-ara	433.1127	317.0655
11	Peo-glc	463.1232	301.0705
12	Mv-gal	493.1339	331.0810
13	Peo-ara	433.1128	301.0705
14	Mv-glc	493.1339	331.0810
15	Mv-ara	463.1231	331.0810
16	D-Hex-Ac	507.1127	303.0490
17	Cy-Hex-Ac	491.1180	287.0548
18	Pet-Hex-Ac	521.1280	317.0655
19	Mv-gal-Ac	535.1444	331.0810
20	Peo-Hex-Ac	505.1330	301.0705
21	Mv-glc-Ac	535.1445	331.0810

D: Delphinidin, Cy: Cyanidin, Pet: Petunidin, Peo: Peonidin, Mv: Malvidin, gal: galactose, glc: glucose, ara: arabinose, Hex: Hexose, Ac: acetate.
