# Supplementary figures and images for: Variation of Anthocyanin Content and Profile Throughout Fruit Development and Ripening of Highbush Blueberry Cultivars Grown at Two Different Altitudes
Source: Front Plant Sci. 2019 Sep 4;10:1045. doi: 10.3389/fpls.2019.01045 (PMC6737079; doi:10.3389/fpls.2019.01045)

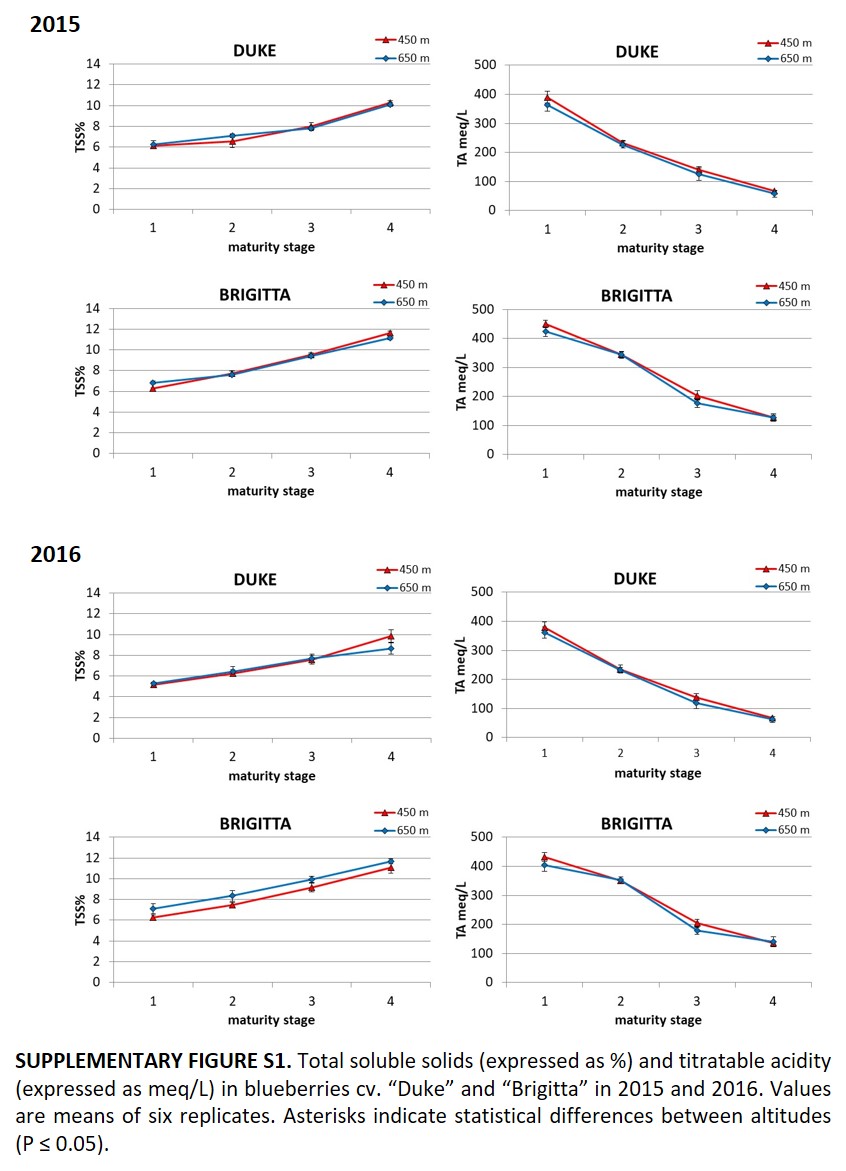

Supplement: Supplementary file 2 [file Image_1.jpg]

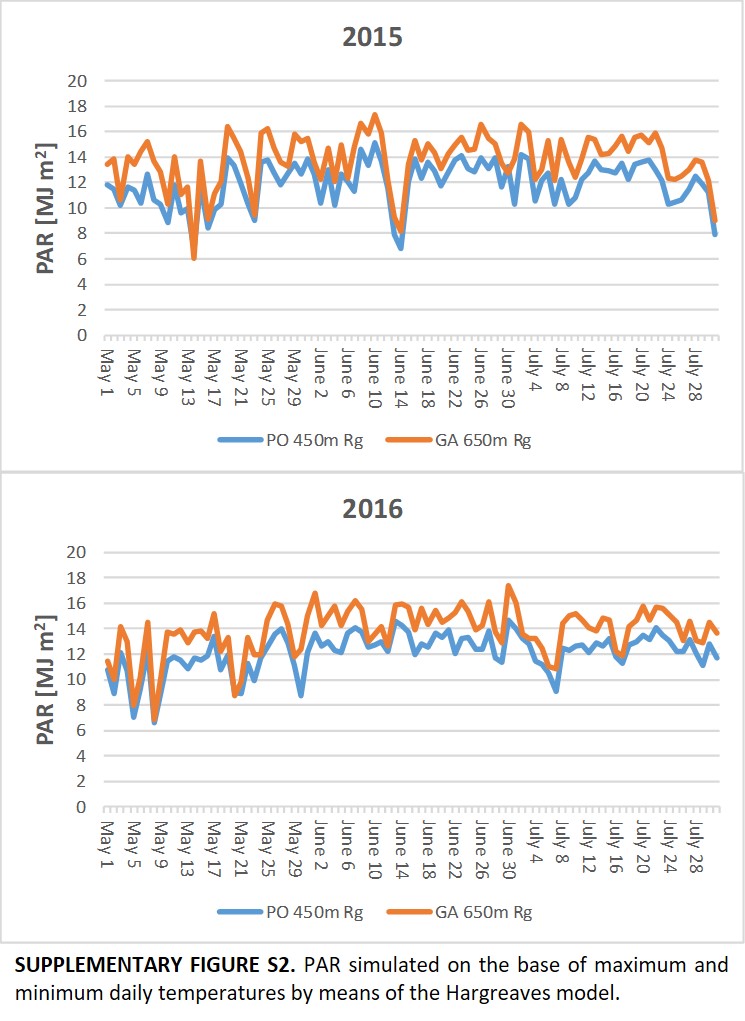

Supplement: Supplementary file 3 [file Image_2.jpg]
