# Supplementary material for: Variation of Anthocyanin Content and Profile Throughout Fruit Development and Ripening of Highbush Blueberry Cultivars Grown at Two Different Altitudes
Source: Front Plant Sci. 2019 Sep 4;10:1045. doi: 10.3389/fpls.2019.01045 (PMC6737079; doi:10.3389/fpls.2019.01045)
Supplement: Supplementary file 4 [file Image_3.pdf]

**SUPPLEMENTARY FIGURE S3.** Chromatogram integrated at 520 nm of Brigitta blueberry fruit.

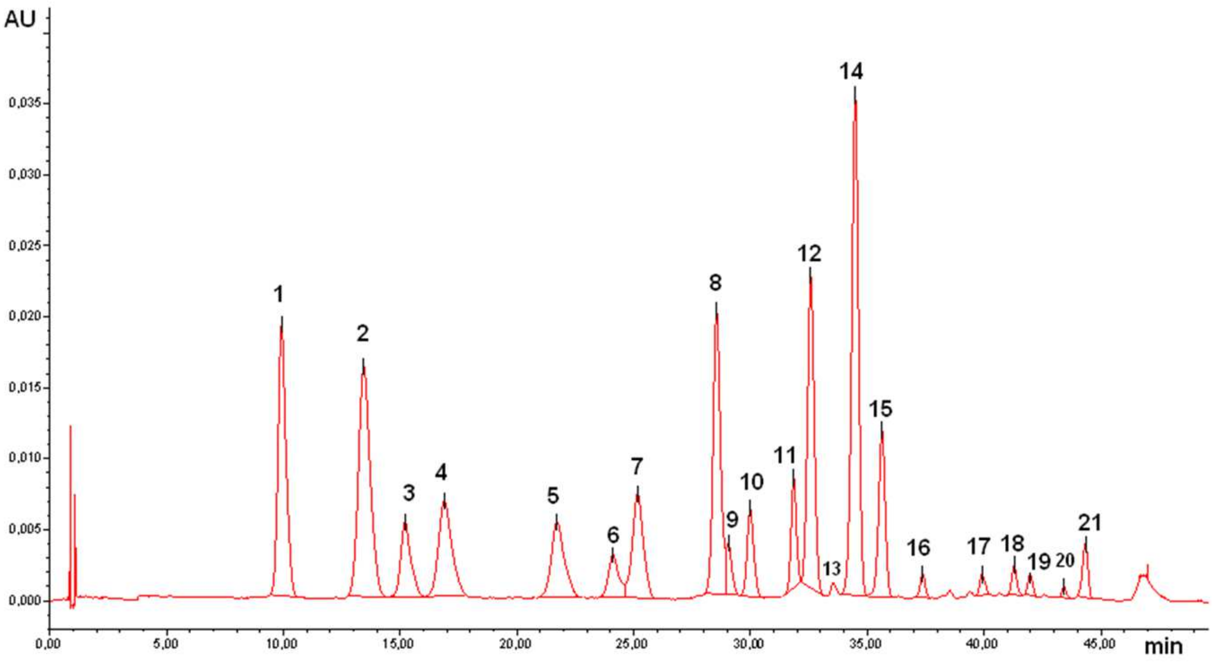


| Peak | Anthocyanin |
|---|---|
| 1 | D-gal |
| 2 | D-glc |
| 3 | Cy-gal |
| 4 | D-ara |
| 5 | Cy-glc |
| 6 | Cy-ara |
| 7 | Pet-gal |
| 8 | Pet-glc |
| 9 | Peo-gal |
| 10 | Pet-ara |
| 11 | Peo-glc |
| 12 | Mv-gal |
| 13 | Peo-ara |
| 14 | Mv-glc |
| 15 | Mv-ara |
| 16 | D-Hex-Ac |
| 17 | Cy-Hex-Ac |
| 18 | Pet-Hex-Ac |
| 19 | Mv-gal-Ac |
| 20 | Peo-Hex-Ac |
| 21 | Mv-glc-Ac |

D: Delphinidin, Cy: Cyanidin, Pet: Petunidin, Peo: Peonidin, Mv: Malvidin, gal: galactose, glc: glucose, ara: arabinose, Hex: Hexose, Ac: acetate.
